# New Proof-of-Concept in Viral Inactivation: Virucidal Efficacy of 405 nm Light Against Feline Calicivirus as a Model for Norovirus Decontamination

**DOI:** 10.1007/s12560-016-9275-z

**Published:** 2016-12-31

**Authors:** Rachael M. Tomb, Michelle Maclean, John E. Coia, Elizabeth Graham, Michael McDonald, Chintamani D. Atreya, Scott J. MacGregor, John G. Anderson

**Affiliations:** 1 The Robertson Trust Laboratory for Electronic Sterilisation Technologies (ROLEST), Department of Electronic & Electrical Engineering, University of Strathclyde, Royal College Building, 204 George Street, Glasgow, G1 1XW Scotland, UK; 2 Department of Biomedical Engineering, University of Strathclyde, Wolfson Centre, 106 Rottenrow, Glasgow, Scotland, UK; 3 Department of Clinical Microbiology, Glasgow Royal Infirmary, Glasgow, Scotland, UK; 4 School of Veterinary Medicine, College of Medical, Veterinary and Life Sciences, University of Glasgow, Glasgow, Scotland, UK; 5 Office of Blood Research and Review, Center for Biologics Evaluation and Research, Food and Drug Administration, Bethesda, MD USA

**Keywords:** 405 nm Light, Viral inactivation, Feline calicivirus, Saliva, Faeces, Plasma

## Abstract

The requirement for novel decontamination technologies for use in hospitals is ever present. One such system uses 405 nm visible light to inactivate microorganisms via ROS-generated oxidative damage. Although effective for bacterial and fungal inactivation, little is known about the virucidal effects of 405 nm light. Norovirus (NoV) gastroenteritis outbreaks often occur in the clinical setting, and this study was designed to investigate potential inactivation effects of 405 nm light on the NoV surrogate, feline calicivirus (FCV). FCV was exposed to 405 nm light whilst suspended in minimal and organically-rich media to establish the virucidal efficacy and the effect biologically-relevant material may play in viral susceptibility. Antiviral activity was successfully demonstrated with a 4 Log_10_ (99.99%) reduction in infectivity when suspended in minimal media evident after a dose of 2.8 kJ cm^−2^. FCV exposed in artificial faeces, artificial saliva, blood plasma and other organically rich media exhibited an equivalent level of inactivation using between 50–85% less dose of the light, indicating enhanced inactivation when the virus is present in organically-rich biologically-relevant media. Further research in this area could aid in the development of 405 nm light technology for effective NoV decontamination within the hospital environment.

## Introduction

Norovirus (NoV), one of the most common causes of epidemic acute gastroenteritis (Hall et al. 2013), can be transmitted via food and water, person-to-person contact or contact with environmental surfaces (Robilotti et al. 2015). Environmental stability and resistance to disinfection further aid the transmission of NoV, with viral particles detected on surfaces up to 42 days after contamination (Escudero et al. 2012). If environmental decontamination is deficient, this can lead to ward closures which has substantial operational and financial implications for health boards (Wu et al. 2005; Danial et al. 2011). NoV outbreaks in the healthcare setting and other densely populated areas such as nursing homes, schools and restaurants (Robilotti et al. 2015) have driven the need for new decontamination systems.

Advanced decontamination technologies used to overcome nosocomial outbreaks include ozone, hydrogen peroxide vapour and UV-light systems (Maclean et al. 2015). These technologies are time consuming with hospital wards required to be vacated to prevent harmful effects to patients and staff (Otter et al. 2013), and are therefore suited to terminal cleaning. A technology using 405 nm violet-blue visible light has been developed to provide continuous decontamination of occupied hospital environments (Maclean et al. 2014). Application of 405 nm light for decontamination in hospitals has been demonstrated, with levels of bacterial contamination on environmental surfaces around occupied isolation rooms reduced by up to 86% over and above reductions achieved by traditional cleaning alone (Maclean et al. 2010, 2013a; Bache et al. 2012).

It has been demonstrated that 405 nm violet-blue light has germicidal activity against a range of bacteria and fungi (Guffey and Wilborn 2006; Enwemeka et al. 2008; Maclean et al. 2009, 2013b; Murdoch et al. 2013), effected through excitation of endogenous photosensitive porphyrin molecules within microbial cells, causing the production of singlet oxygen and other reactive oxygen species (ROS), resulting in oxidative damage and microbial cell death (Hamblin and Hasan 2004; Maclean et al. 2008; Murdoch et al. 2013). A study investigating the efficacy of 405 nm light on the bacteriophage ϕC31 indicated that the phage was susceptible to high doses of 405 nm light, with susceptibility significantly enhanced when exposed in nutrient-rich media (Tomb et al. 2014). However, as virions do not contain endogenous porphyrins (Gelderblom 1996), current knowledge on the antiviral efficacy of 405 nm light on medically important human and animal viruses is lacking and requires investigation.

This study was designed to provide the first proof-of-concept of the interaction of narrowband 405 nm light with feline calicivirus (FCV) as a model to study the antiviral effects of this light on NoV. Feline calicivirus was selected as a NoV surrogate, as there is currently no standardised cell culture system for NoV (Duizer et al. 2004a; Richards 2012; Cromeans et al. 2014). Our data demonstrate the influence of the suspending media, including biologically-relevant fluids, on viral susceptibility. As such, this study provides evidence of the antiviral efficacy and discusses the potential mechanism of 405 nm light viral inactivation.

## Methodology

### Cell and Virus Culture

Feline embryonic cells, strain FEA (Jarrett et al. 1973), were cultured in Dulbecco’s modified eagle’s medium (DMEM) supplemented with 10% foetal bovine serum (FBS), 2 mM l-glutamine, 1 mM sodium pyruvate and 240 U mL^−1^ penicillin streptomycin (Gibco, Life Technologies, UK), to form 10% FBS-DMEM. Cells were maintained at 37 °C in 5% CO_2_.

To prepare a virus pool of the FCV vaccine strain F9, virus inoculum (School of Veterinary Medicine, University of Glasgow) was added to FEA monolayers in 850 cm^2^ cell culture roller flasks (Corning, USA). After 90 min incubation of the inoculated cells on a rotating roller stand at 37 °C in 5% CO_2_, fresh culture medium was added and flasks incubated for 24 h. This resulted in virus-induced destruction of nearly 90% of the cell monolayer.

The tissue culture supernatant, and medium from a single wash step, was collected from each roller bottle and subjected to two freeze–thaw cycles before clarification by centrifugation at 3300×*g* for 10 min. The virus-containing supernatant was then stored at −80 °C until required. The infectious titre of FCV was approximately 2 × 10^7^ plaque-forming units per millilitre (PFU mL^−1^), determined by standard plaque assay techniques (Ormerod and Jarret 1978).

### 405 nm Light Source

**Fig. 1:**
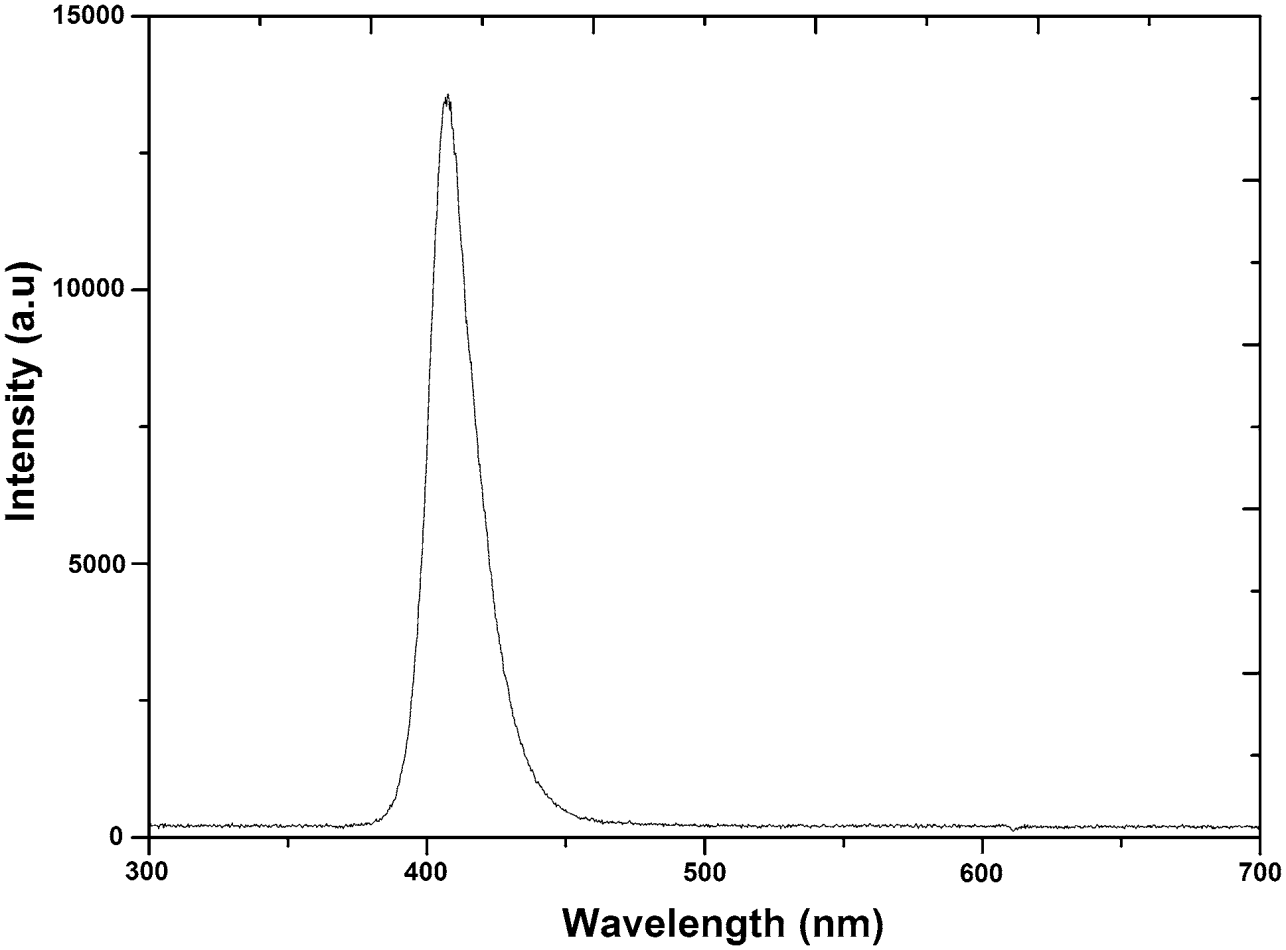
Optical emission spectrum of the 405 nm LED array, measured using an HR4000 spectrometer (Ocean Optics, Germany) and Spectra Suite software version 2.0.151

The light source used was a 405 nm light emitting diode (LED) array (ENFIS PhotonStar Innovate UNO 24; PhotonStar Technologies, UK) powered by a 40 V Phillips Xitanium LED Driver (Phillips, Netherlands). The array had a peak wavelength around 405 nm and a bandwidth of approximately 19 nm (Fig. 1) but will, for convenience, be referred throughout this text as 405 nm light. The array was attached to a heatsink and cooling fan, to minimise heat transfer to test samples, so that no significant heating of the sample occurred. The light source was held on a PVC stand at a distance of 4 cm from the microbial samples, giving an irradiance of 155.8 mW cm^−2^ at the sample surface [measured using a radiant power meter and photodiode detector (LOT Oriel, USA)].

### 405 nm Light Exposure of Viral Suspensions

Feline calicivirus stock virus was defrosted at room temperature and diluted to 2 × 10^5^ PFU mL^−1^ in Dulbecco’s phosphate-buffered saline, supplemented with calcium and magnesium (DPBS; Hyclone, Thermo Fischer Scientific, UK). This was used as a ‘minimal medium’ (MM). Viral suspension of 1.5 mL were transferred into the central four wells of a 24-well plate (Techno Plastic Products, Switzerland) and the plate positioned on a raised stand, with the sample wells at 4 cm directly below the light source and the plate lid kept on to prevent evaporation. Test samples were exposed to increasing doses of 405 nm light at room temperature, with the dose calculated as the product of irradiance (mW cm^−2^) × exposure time (s). Control samples were set up under identical environmental conditions but without 405 nm light illumination. Post-exposure, FCV samples were serially diluted in MM for enumeration by plaque assay.

Exposures were repeated with FCV suspended in ‘organically-rich media’ (ORM): DMEM, 10% FBS-DMEM, artificial saliva, artificial faeces and blood plasma. The artificial saliva was a modified version of that used by Margomenou et al. (2000) [5.2 g NaHCO_3_, 0.88 g NaCl, 1.36 g K_2_HPO_4_, 0.48 g KCl, 2000 units α-amylase and 2 g pig gastric mucin (Sigma-Aldrich, UK) in 1 L sterile water], and was adjusted to pH of 7–7.5 to emulate the variability of pH in human saliva, and also to ensure that no FCV inactivation occurred (Duizer et al. 2004b; Edgar et al. 2004). The artificial faeces was a modified version of that by Colón et al. (2015) [30 g inactivated yeast (Marigold, UK), 7 g physillum (Buy Whole Foods Online, UK), 11 g miso paste (Yutaka, UK), 8 g cellulose, 1.6 g NaCl, 0.8 g CaCl, 1.6 g KCl (Sigma-Aldrich) in 920 mL sterile water], and was also adjusted to pH 7. The modifications to the formulations of artificial saliva and faeces were to ensure compatibility with the FEA cells. Fresh frozen human blood plasma was obtained from the Scottish National Blood Transfusion Service (SNBTS, UK), and defrosted before use. FCV was also exposed when suspended in MM supplemented with riboflavin, with and without tyrosine, tryptophan, pyridoxine and folic acid (used at the same concentrations as found in DMEM: 0.4, 104, 16, 4 and 4 mg L^−1^ respectively).

### Plaque Assay

Prior to experiments, 6-well cell culture plates (Thermo Fischer Scientific) were seeded with 7.5 × 10^5^ FEA cells per well. 3 mL of the cell suspension in growth medium was pipetted into each well, and incubated at 37 °C in 5% CO_2_ for 20 h, resulting in confluent monolayers.

Post-exposure of FCV, the growth medium was aspirated from the FEA cells and replaced with 1 mL FCV sample. Plates were co-incubated at 37 °C in a humidified 5% CO_2_ incubator for 90 min, with the plates gently rocked every 15 min to ensure even distribution of the inoculum over each monolayer.

After the viral incubation period, the inoculum was aspirated and the well washed with medium (10% FBS-DMEM or DPBS) before adding 4 mL overlay mixture consisting of 2× supplemented DMEM 1:1 with 2× agarose. 2× supplemented DMEM was prepared using 20 mL from a filter-sterilised stock of 10× DMEM, adding the same supplements as detailed earlier, plus 9.86 mL sodium bicarbonate solution (Gibco), and was made up to 100 mL with sterile water. 2× agarose was prepared by dissolving 2 g agarose (Sigma-Aldrich) in 100 mL deionised distilled water and then sterilised by autoclaving. The overlay was left to set before the plates were incubated for 44–48 h at 37 °C in 5% CO_2_.

Post-incubation, the monolayers were fixed and stained overnight with 0.5% crystal violet in 10% neutral buffered formalin. The agarose plugs and stain were then removed, the plates left to dry, plaques counted, and the virus infectivity titre expressed as PFU mL^−1^.

### Spectrophotometry

The transmission of 405 nm light through the suspending media was measured using a Biomate 5-UV–Visible spectrophotometer (Thermo Fischer Scientific). The presence of porphyrins, or other components with the ability to absorb 405 nm light and emit fluorescence, within the suspending media was determined by fluorescence spectrophotometry. Media were freshly prepared, and fluorescence measurements were carried out using a RF-5301 PC spectrofluorophotometer (Shimadzu, USA). Excitation was carried out at 405 nm and emission spectra recorded between 425 and 700 nm.

### Data Analysis

Data points represent mean results ± standard deviation (SD), taken from triplicate independent experiments (*n* = ≥3). The antiviral activity of 405 nm light was determined by calculating the reduction in the level of infectivity from the difference between Log_10_ values for exposed and control samples. Significant differences were calculated at a 95% significance level, using paired *t*-tests or one-way ANOVA (Minitab 16 Statistical Software), with results found to be significant when *P* < 0.05.

## Results

**Fig. 2:**
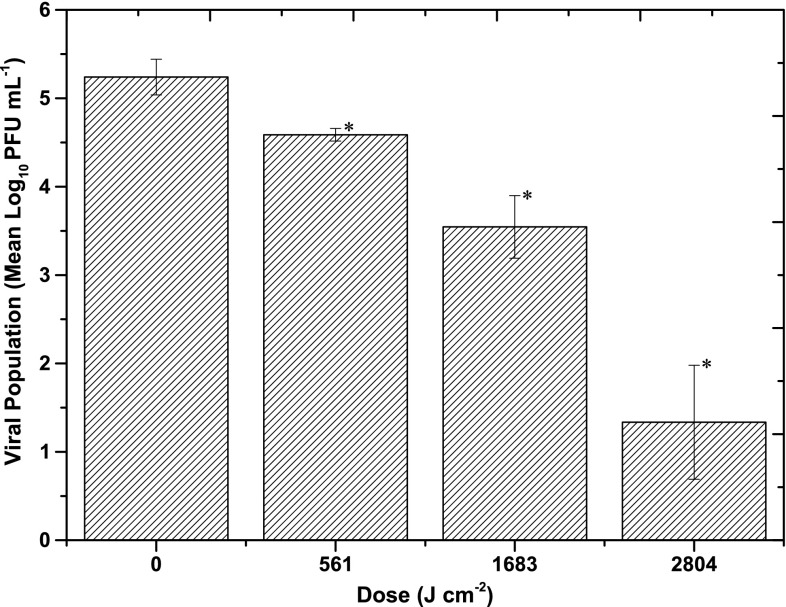
Inactivation of feline calicivirus when suspended in minimal medium (Dulbecco’s phosphate buffered saline), upon exposure to 405 nm light at an irradiance of 155.8 mW cm^−2^. Data points show the mean counts (*n* = 6) ± SD. *Asterisks* indicate light-exposed samples that were significantly different to the non-exposed final control samples (*P* ≤ 0.05), using one-way ANOVA. No significant decrease was observed in the final control populations (*P* ≥ 0.05)

Feline calicivirus was suspended in MM and ORM and exposed to increasing doses of 405 nm light at an irradiance of 155.8 mW cm^−2^. Results (Fig. 2) show that when suspended in MM, significant FCV inactivation was achieved after exposure to 561 J cm^−2^ (*P* = 0.043), and relatively linear inactivation kinetics were observed, with a dose of 2.8 kJ cm^−2^ required for a 3.9 Log_10_ inactivation. The non-exposed control samples showed no significant change over the course of the experiment (*P* > 0.05).

**Fig. 3:**
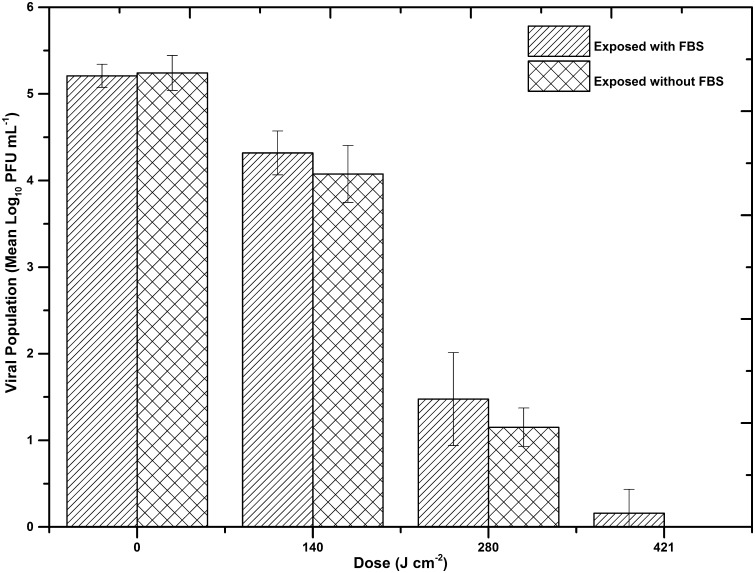
Comparison of the inactivations of feline calicivirus when suspended in organically-rich media [supplemented Dulbecco’s modified eagle’s medium, without and without 10% fetal bovine serum (FBS)], upon exposure to 405 nm light at an irradiance of 155.8 mW cm^−2^. Data points show the mean counts (*n* = 3) ± SD. Statistical analysis, using a paired *t* test, showed no significant difference between inactivations in the two media (*P* > 0.05). No significant decrease was observed in the final control populations (*P* ≥ 0.05)

Antiviral efficacy was found to differ significantly when suspended in ORM. When exposed in 10% FBS-DMEM, a significantly lower dose was required for viral inactivation (Fig. 3), with a 4.8 Log_10_ reduction achieved after a dose of 421 J cm^−2^. As the presence of FBS in DMEM is thought to reduce the level of oxidation upon exposure to normal laboratory lighting (Grzelak et al. 2001), the exposure was repeated with FCV suspended in DMEM without FBS to observe any differences in inactivation kinetics. Although slightly greater inactivation was observed with each applied dose, results (Fig. 3) demonstrate no significant differences in the inactivation kinetics of FCV when the virus is exposed in DMEM in the presence or the absence of 10% FBS (*P* > 0.05). Control samples showed no significant decrease (*P* > 0.05).

Furthermore, components of DMEM have been shown to be photosensitive to light (Grzelak et al. 2001), and therefore, exposures were repeated with riboflavin added to MM with and without tyrosine, tryptophan, pyridoxine and folic acid in the same concentrations as found in DMEM (Table 1). Results demonstrated that exposure of FCV suspended in MM with riboflavin only resulted in a 1.3 Log_10_ reduction after 421 J cm^−1^; however, when all components were present, enhanced inactivation occurred and a 5.1 Log_10_ inactivation was achieved.

**Table 1:** Comparison of the inactivations of feline calicivirus when suspended in minimal media supplemented with riboflavin alone or alongside tyrosine, tryptophan, pyridoxine and folic acid, upon exposure to 405 nm light at an irradiance of 155.8 mW cm^−2^

Photosensitive components	Starting population, Log_10_ PFU mL^−1^ (± SD)	Exposed viral population, Log_10_ PFU mL^−1^ (± SD)	Non-exposed control population, Log_10_ PFU mL^−1^ (± SD)	Log_10_ reduction, PFU mL^−1^ (*P* value)
Riboflavin	5.01 ± 0.02	3.77 ± 0.61	5.05 ± 0.06	1.28* (*P* = 0.00)
Riboflavin Tyrosine Tryptophan Pyridoxine Folic acid	5.15 ± 0.03	0.00 ± 0.00	5.12 ± 0.07	5.12* (*P* = 0.00)

Data points represent the mean count (*n* = 3) ± SD

* Light-exposed samples that were significantly different to the non-exposed final control samples (*P* ≤ 0.05)

**Fig. 4:**
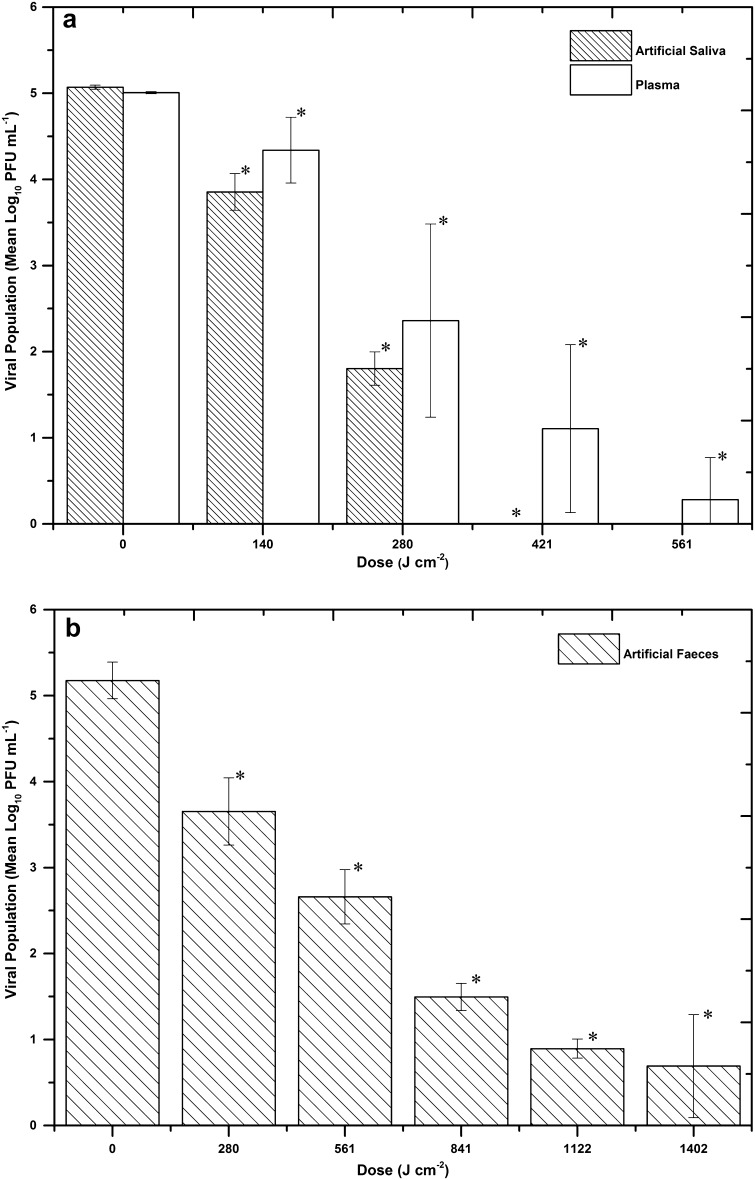
Inactivation of feline calicivirus suspended in **a** artificial saliva or plasma and **b** artificial faeces, upon exposure to increasing doses of 405 nm light at an irradiance of 155.8 mW cm^−2^. Data points show the mean counts (*n* = 3) ± SD. *Asterisks* indicate light-exposed samples that were significantly different to non-exposed final control samples (*P* ≤ 0.05), using one-way ANOVA. No significant decrease was observed in the final control populations (*P* ≥ 0.05)

Artificial saliva, artificial faeces and blood plasma were selected as ORM which are biologically relevant in terms of media in which viral particles may be found in the environment, with NoV being regularly identified in faeces. Exposure of FCV when suspended in artificial saliva yielded results similar to those in DMEM, with a 5.1 Log_10_ reduction of infectivity achieved after a dose of 421 J cm^−2^ (Fig. 4a). (In this case, inactivation was measured to a sensitivity of ten PFU mL^−1^, as the artificial saliva in the undiluted samples adversely reacted with the FEA cells causing them to dislodge from the plate). The dose required for inactivation when suspended in blood plasma was slightly greater than that required when in artificial saliva, with 561 J cm^−2^ being required for 4.8 log_10_ inactivation of FCV (Fig. 4a). FCV inactivation in artificial faeces required greater doses, with 4.5 log_10_ inactivation achieved after 1.4 kJ cm^−2^ (Fig. 4b). Control samples in artificial saliva, plasma and artificial faeces showed no significant changes (*P* = 0.618, 0.101, 0.747, respectively).

**Fig. 5:**
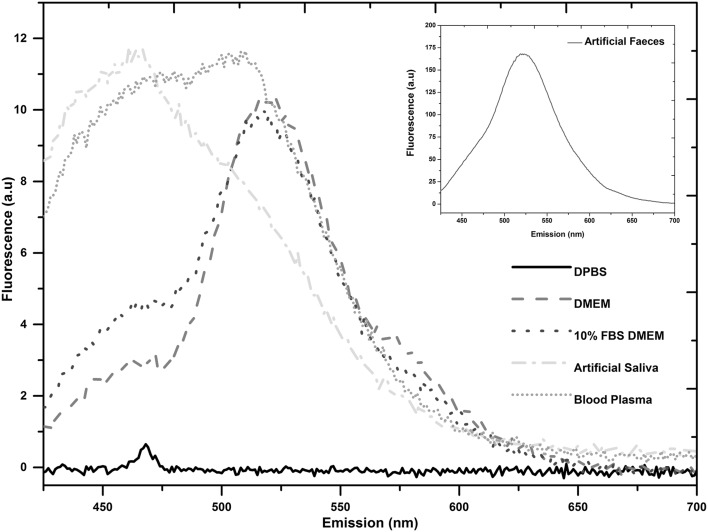
Fluorescence spectra of minimal medium [Dulbecco’s phosphate buffered saline (DPBS)] and organically-rich media [Dulbecco’s modified eagle’s medium (DMEM), 10% foetal bovine serum-supplemented DMEM (10% FBS-DMEM), artificial saliva, artificial faeces and blood plasma] using an excitation wavelength of 405 nm

Optical analysis of the suspending media demonstrated the transmission of 405 nm light to be 90% in DPBS, 40.6% in DMEM, 30.6% in 10% FBS-DMEM, 35.9% in artificial saliva, 0.05% in artificial faeces, and 2.1% in blood plasma (*n* = 4). The fluorescence emission spectra (Fig. 5) of MM (DPBS) and ORM (DMEM, 10% FBS-DMEM, artificial saliva, artificial faeces and blood plasma) when excited at 405 nm, show emission peaks for DMEM, 10% FBS-DMEM, artificial faeces and blood plasma observed between 510 and 520 nm and for artificial saliva at 460 nm.

## Discussion

Although there has been a recent move towards using Murine Norovirus and Tulane Virus, alongside FCV, as NoV surrogates (Cromeans et al. 2014; Kniel 2014; Chui et al. 2015; Esseili et al. 2015; Zonta et al. 2016), FCV was chosen as it has physiochemical and genomic similarities to NoV, and is a well-established surrogate with a standardised cell culture protocol (Doultree et al. 1999; Bidawid et al. 2003; Duizer et al. 2004a, 2004b; Chander et al. 2012). Similarly, studies investigating the virucidal effects of UV-light, ozone, hydrogen peroxide vapour and cold atmospheric gas plasma technologies have also used FCV as a NoV surrogate (Nuswalen et al. 2002; Hudson et al. 2007; Bentley et al. 2012; Aboubaktar et al. 2015; Holmdahl et al. 2016).

The virucidal efficacy of 405 nm light was determined using FCV suspended in both MM and ORM. Exposure in MM would provide a better indication of the interaction of 405 nm light and the virus alone, when under suspension in ORM, which is likely to contain photosensitive components, and would assess how viral susceptibility can potentially be influenced by the surrounding media.

Successful FCV inactivation was achieved when suspended in MM, although the dose required was substantially great, with 2.8 kJ cm^−2^ achieving a 3.9 Log_10_ reduction (Fig. 2). In the case of bacteria and fungi in MM, doses in the range of 18–576 J cm^−2^ are typically required for 5 Log_10_ inactivations (Maclean et al. 2009; Murdoch et al. 2012, 2013). The increased susceptibility of bacteria and fungi compared with viruses is accredited to the presence of endogenous photosensitive porphyrins within these cells (Hamblin and Hasan 2004; Maclean et al. 2008; Murdoch et al. 2013). Low sensitivity of FCV in MM was anticipated due to the absence of porphyrins in the viral structure, coupled with the fact that MM does not contain any photosensitive substances which absorb light at 405 nm (Fig. 5), suggesting that viral inactivation, in this case, is due to a differing mechanism.

An alternative mechanism of inactivation when FCV is suspended in MM may be associated with the LED emission spectrum extending slightly into the UVA region (Fig. 1), meaning the virus is exposed to very low-level UVA photons (~390 nm). Over an extended period, this could cause oxidative damage to proteins (Girard et al. 2011), for example, to the viral capsid, and therefore contribute to the observed inactivation. Another possibility is that the small amount of 420–430 nm light emitted from the source may contribute to viral inactivation. Antiviral effects of 420–430 nm have been demonstrated against murine leukaemia virus, with long exposures thought to cause photo-damage to the virion-associated reverse transcription complex (Richardson and Porter 2005). Although the virus differs in structure to FCV, these findings suggest that the prolonged exposure to wavelengths at the tail ends of the 405 nm LED emission spectrum such as 390 and 420 nm, as well as 405 nm, may affect the viruses’ ability to infect and replicate in host cells, and have a role in the inactivation of FCV by the LEDs used in this study.

To investigate whether exposure in ORM had any effect on viral susceptibility, FCV was first suspended in DMEM with and without 10% FBS, thought to aid protection against ROS (Grzelak et al. 2001). Results (Fig. 3) demonstrated near complete reduction in infectivity of a 10^5^ PFU mL^−1^ population after a dose of 421 J cm^−2^. As can be seen in Fig. 3, slightly greater inactivation occurred when FCV was suspended in DMEM without the FBS serum additive; however, no significant difference was seen between the inactivation kinetics. As the inactivation dose of 421 J cm^−2^ is 85% less than that required for a similar level of inactivation in MM, it is likely that components of the ORM are influencing FCV inactivation. A study investigating the susceptibility of bacteriophage ϕC31 (Tomb et al. 2014) demonstrated similar results to those of the current study: little inactivation was observed when exposed in a simple salt solution; however, susceptibility was significantly enhanced when suspended in a nutrient-rich medium, with a 5.4 Log_10_ reduction of ϕC31 achieved after exposure to 510 J cm^−2^. This was hypothesised to be due to the complex protein and amino acid-rich composition of the nutrient-rich medium, suggesting that some components could be photosensitive and when exposed to 405 nm light in the presence of oxygen, would produce ROS, damaging the bacteriophage (Tomb et al. 2014). This same phenomenon is likely to account for the enhanced inactivation of FCV when suspended in DMEM and 10% FBS-DMEM, as these contain a complex mixture of amino acids, vitamins and sugar, which have the potential to absorb 405 nm light (Fig. 5) and act as photosensitisers.

The photosensitisation of components of DMEM has also been demonstrated upon exposure to light, with riboflavin being shown to produce ROS which is further enhanced by tryptophan, tyrosine, pyridoxine and folic acid (Grzelak et al. 2001). Furthermore, blue-light wavelengths are thought to be the most efficient for the photo-decomposition of riboflavin and generation of ROS (Cheng et al. 2015). To investigate this, riboflavin was added to MM with and without tyrosine, tryptophan, pyridoxine and folic acid in the same concentrations found in DMEM (Table 1). Results support this, with only 1.3 Log_10_ reduction when only riboflavin was present; however, when all vitamins and amino acids (riboflavin, tyrosine, tryptophan, pyridoxine and folic acid) were present, enhanced inactivation of FCV was achieved with complete inactivation of a 10^5^ PFU mL^−1^ population.

It is important to consider how light-induced inactivation would be influenced when viral particles were suspended in more biologically-relevant, naturally occurring matrices such as body fluids or secretions. As artificial saliva and artificial faeces can be prepared, these were used alongside human blood plasma, as model human secretions in which many viruses can be transmitted (Aitken and Jeffries 2001).

Results (Fig. 4) demonstrated that, similar to inactivation in ORM (DMEM and 10% FBS-DMEM), viral susceptibility was significantly increased when suspended in these biologically-relevant fluids. Of the three model fluids used, sensitivity was the highest when suspended in artificial saliva, with a 5.1 Log_10_ reduction of FCV infectivity being achieved after a dose of 421 J cm^−2^—the same as that observed when suspended in ORM. Susceptibility was slightly reduced when suspended in blood plasma (4.8 Log_10_ inactivation with 561 J cm^−2^), and further reduced when suspended in artificial faeces, with more than three times the dose required to achieve a 4.5 Log_10_ reduction. The reduced levels of 405 nm light transmission through the blood plasma and artificial faeces will contribute to these slower inactivation rates, with average values of 2.12 and 0.05% transmission levels of 405 nm recorded for blood plasma and artificial faeces, respectively, compared to 30–40% transmission levels in all other ORM used. Overall, the susceptibility values of FCV to 405 nm light when suspended in artificial faeces, artificial saliva, blood plasma and other organically rich media were significantly increased compared to the susceptibility in minimal media, with 50–85% less dose being required for similar levels of viral inactivation. Inactivation when suspended in these ORM is likely due to the proteins contained within the media, for example, the mucin in the artificial saliva, proteins within the plasma, and inactivated yeast within the artificial faeces, which may all be predisposed to photosensitisation (demonstrated by the fluorescence peaks around 460 and 510–520 nm in Fig. 5). These results indicate the potential for NoV susceptibility to 405 nm light to be enhanced when suspended in ORM, or host secretions in which they are released, such as faeces, blood and vomit. Although the consistency and transparency/opacity may differ to those used in this study, these fluids are likely to be rich in molecules which could potentially be sensitive to 405 nm light, thereby aiding in the NoV inactivation.

The results of this study provide first proof-of-concept demonstrating that the antimicrobial efficacy of 405 nm light can be extended to medically important viruses, with the susceptibility being significantly enhanced when the viral particles are contained within biologically-relevant media. Further work should be carried out to establish the effects of 405 nm light on other NoV surrogates, such as Murine Norovirus and Tulane Virus, which may be more resistant to decontamination. This will ensure that the antiviral efficacy of 405 nm light is not over/under-estimated and allows for a more accurate quantification of the dose required for NoV inactivation. In addition, as this work used a small-scale LED source with a high irradiance output to establish the inactivation kinetics, and further investigations are therefore required to investigate the effectiveness of 405 nm light against airborne and surface-deposited viruses, using low irradiance light applied continuously over long periods, similar to that employed in clinical decontamination evaluations (Maclean et al. 2010, 2013a; Bache et al. 2012). Further studies could lead to the beneficial application of 405 nm light for the decontamination of air, surfaces and equipment in healthcare settings, as well as in other indoor locations, where transmission of viral pathogens is a significant occurrence.
